# Scan Aided Dental Arch Width Prediction via Internationally Recognized Formulas and Indices in a Sample of Kurdish Population/Iraq

**DOI:** 10.3390/diagnostics13111900

**Published:** 2023-05-29

**Authors:** Trefa Mohammed Ali Mahmood, Arass Jalal Noori, Zana Hussein Aziz, Aras Maruf Rauf, Fadil Abdulla Kareem

**Affiliations:** 1 Department of Pedodontics, Orthodontics and Preventive Dentistry, College of Dentistry, University of Sulaimani, Sulaimaniyah 46001, Iraq; arass.noori@univsul.edu.iq (A.J.N.); aras.rauf@univsul.edu.iq (A.M.R.); fadil.kareem@univsul.edu.iq (F.A.K.); 2 Sulaimani Health Directory, Sulaimani University Medical Center, Sulaimani 46001, Iraq; drzana78@yahoo.com

**Keywords:** anterior and posterior arch widths, Pont’s index, correlation, Kurdish sample

## Abstract

**Background**: Numerous studies have investigated the applicability of Pont’s index using a variety of selection criteria. The morphology of teeth and the shapes of the face are significantly influenced by racial, cultural, and environmental factors, so the current study focused on these demographics. **Methods**: This study is a retrospective study and included one hundred intraoral scanned images selected from patients seeking orthodontic treatment. Medit design software was used to obtain the real measurements and compare them to the predicted values from Pont’s index. Paired t tests were used to test the validity of Pont’s index, and regression equations were advocated to predict the inter-molar, inter-premolar, and anterior arch widths via SPSS version 25. **Results**: There were significant differences between the real anterior, inter-premolar, and inter-molar widths and the predicted values obtained from Pont’s index, and there were weak positive correlations between the real values and the predicted values from Pont’s index. **Conclusions:** Pont’s index is not reliable to predict the arch widths for the Kurdish population, and new formulas are advocated. Hence, space analysis, malocclusion treatment, and arch expansion therapy should all take into account these results. Therefore, the derived equations may have further positive effects on diagnoses and treatment preparation.

## 1. Introduction

One of the fundamentals of orthodontic diagnosis and treatment plan construction is dental arch dimension analysis [1]. In orthodontics, various model analytical indices have been presented to aid in diagnoses and treatment planning. The majority of malocclusions can be corrected using either extraction or non-extraction techniques. Proximal stripping, distalization, labialization, and arch expansion are all non-extraction methods. Among these scenarios, expansion is the most commonly recommended, but its relapse has long been called into question [2]. Clinicians can forecast the probable arch width due to the availability of several indices [3,4,5]. All of these indices reveal a relationship between the maxillary incisors’ arch length, arch breadth, and mesiodistal width. Therefore, many attempts have been made in order to find correlations between tooth size and various arch dimensions, including arch width and arch length [1]. In 1900, Pont proposed an equation that predicts the anterior, premolar, and molar arch width based on the summation of the mesiodistal dimensions of the maxillary incisors (SUI) by formulating different equations [3]. Pont recommended investigating his proposed formula in populations other than French ones so as to test its reliability because he was aware of the possibility of differences between different ethnic groups. The potential benefits of using Pont’s Index in diagnostics and treatment planning [6] may stem from the fact that it is simple to implement. Its accuracy in estimating dental arch widths has been supported by some studies [7,8]. In spite of the fact that some researchers, like Lohakare, have concluded that Pont’s index should be used to predetermine ideal arch width values and applied clinically for patients undergoing orthodontic and other dental treatment [8], others have concluded that the index is not reliable enough to be used for clinical purposes [9,10,11,12] due to the fact that its dimension is affected by ethnicity [13], nutrition [14], systemic diseases [15], hormonal factors [16], and gender [17].

When the upper incisors are abnormally large or tiny, the arch width is calculated by adding the widths of the lower incisors (SLI = Sum of Lower Incisors) [16]. The total mesiodistal width of the lower incisors is offered as another index for determining the proper dental arch length [3]. On the basis of his research into palatal depth (PD) measurements, Korkhaus and colleagues (in the year 1900) found a correlation between “posterior arch width” (PAW) and palatal depth in cases of normal occlusion [16].

More academics have taken notice of Pont’s index. Stiften et al. and later Gupta et al. [17] both endorsed this index where complete blockage was the intended outcome of treatment. However, they did admit that there are certain margins for exceptions. Both Joondeph et al. [18] and Dalidjan et al. [9] argued that the clinical utility of Pont’s index is limited. In fact, this area of study is under-researched [12].

Pont proposed that this technique may be used to identify the appropriate dental arch required to accommodate the dentition and relieve crowding, as well as to serve as a guide for expanding the dental arch and a gauge for arch development. The clinical use of premolar and molar indices for determining dental arch growth goals has recently attracted renewed interest [18].

The use of Pont’s index is crucial since it can give important information regarding tooth size variations and associated crowding or spacing problems. Tooth size discrepancies should be diagnosed to determine their impact on orthodontic treatment. Orthodontists can use Pont’s index to determine if a patient has tooth size discrepancies, which can be categorized as either tooth-size excess or tooth-size deficiency. The index gives a quantitative measurement that aids in locating any major departures from the optimal tooth size ratio by comparing the mesiodistal widths of different teeth. Planning the treatment and choosing the best orthodontic strategy both depend on this information [19].

As such, a study has not been conducted to investigate Pont’s index in a Kurdish population. The present study was aimed at the assessment of Pont’s index to predict anterior and posterior arch widths in a sample of the Kurdish population.

## 2. Materials and Methods

### 2.1. Study Registration

Registration and ethical approval were obtained from the ethical committee of the College of Dentistry at the University of Sulaimani (ethical approval number 454 on 14 June 2022) and the study was performed in accordance with the Helsinki Declaration.

### 2.2. Sample

**Figure 1:**
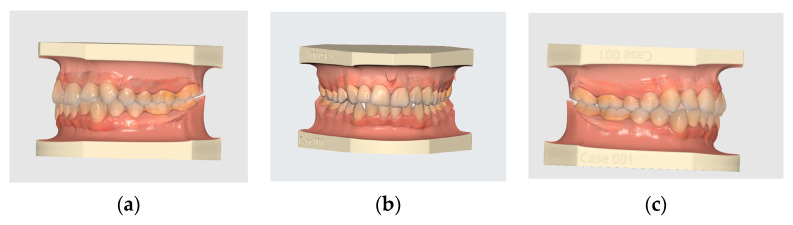
Intra-oral scanned images of one of the selected class I cases (**a**—left buccal side, **b**—right buccal side, **c**—frontal view).

This retrospective study included 100 intraoral scanned (IOS) images (as shown in Figure 1) selected from orthodontic patients seeking treatment in private orthodontic centers. An experienced orthodontist who was specifically educated in and has performed thousands of practice scans using an intraoral scanner (Medit i 700 Wireless) according to the manufacturer’s guidelines acquired all the IOS images. The scanning method was carried out in an upright position in a dental chair for each patient [19]. Medit Design software is a complex tool that can analyze, align, measure (including distance, area, length, and angle), and compare 3D data. It offers a variety of tools to obtain the desired results, including “boolean”, “offset”, “smooth Surface”, “sculpting”, and many others.

The criteria for sample selection include the following (Figure 1):

1. Permanent dentition, class I skeletal and dental relationships, with normal overbite and overjet.
2. Patients’ age is between (18–30) years old.
3. No caries, no attrition, no crossbite, and no crowding of more than 3 mm.
4. No systemic disease, no facial asymmetry, and no congenital disorders.
5. No trauma and no patients under orthodontic treatment.
6. Participants signed a consent form after being contacted via their registered mobile number.

**Figure 2:**
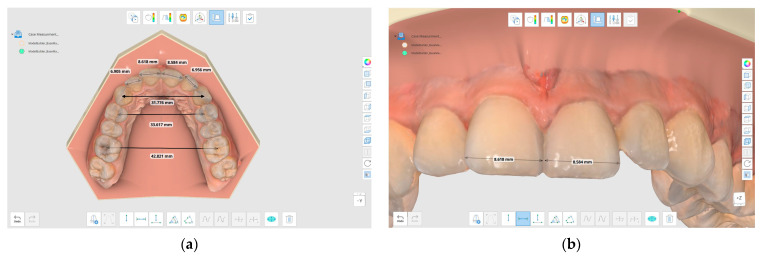
(**a**) The measurement of the IMW, IPW and AAW. (**b**) The mesiodistal dimensions of the upper incisors with Medit design software.

The landmarks (Figure 2) used for measurements were as follows [6]:

1. Anterior Arch Width (AAW): the millimeter distance between the cusp tips of the canines.
2. Inter-premolar width (IPW): the millimeter distance between the distal pits of the maxillary first premolars.
3. Inter-molar width (IMW): the millimeter distance between the central fossae of the maxillary first molars.
4. Mesiodistal dimension of permanent upper incisors (SUI).

Real measurements were obtained from the scanned images and compared with the values obtained from Pont’s equation that predicts anterior, inter-premolar, and inter-molar arch widths based on the summation of the mesiodistal dimensions of maxillary incisors (SUI) using the following equations [3]:

Predicted anterior arch width (AAW) = SUI × 100/85

Predicted inter-premolar arch width (PIP) = SUI × 100/80

Predicted inter-molar width (IMW) = SUI × 100/65

### 2.3. Statistical Analysis

The Shapiro–Wilk test was employed to ensure that the data were normally distributed. On ten randomly selected photos, inter- and intra-examiner calibrations were performed. To evaluate the differences in inter- and intra-examiner calibrations, a paired *t*-test was used. The gathered data were analyzed descriptively and inferentially using the Statistical Package for the Social Sciences (SPSS) software version 25.

The correlation coefficients between observed and expected arch widths were calculated using Pont’s index. The same observer measured all the dimensions. The coefficient of correlation was used to determine the effect of the maxillary incisors’ combined mesiodistal dimensions on the maxillary inter-molar and inter-premolar arch widths.

The Pearson correlation coefficient test was utilized to examine the link between the sum of mesiodistal dimensions of the upper incisors and each dental arch width. The predictor(s) of dental arch widths were discovered using stepwise regression analysis. A paired *t*-test was used to compare the actual and anticipated arch widths (predicted with the new regression equations).

## 3. Results

### 3.1. Sample

Before beginning the main research project, a pilot study of 10 samples was carried out, with 5 males and 5 females chosen at random. This was performed so that the sample size could be estimated. The G*Power program, version 3.1, was used to perform the calculations on the sample size based on a paired *t*-test (power set at 0.8 and the alpha probability error set at 0.05) between the actual measurements of IM, IPre, and IC and the projected values derived using Pont’s equations. From these, the inter-molar measurement showed the lowest effect size (0.8699) with a sample size of 13 per group. Using the IM measurements of a sample of 15, alpha (0.5), and the measurement means and standard deviations from the pilot study, it was found that the power of the statistical tests to obtain a significant result is 0.9484, which is regarded as very high. Furthermore, increasing the sample size to 100 ensures more accurate results that can be safely translated to population parameters. Therefore, it was determined that less than 15 samples were needed to find a statistically significant difference in any of the metrics. To ensure that the results are as accurate as possible and that the sample is as representative of the population as a whole as possible, it was decided that the sample should be large (100 people), and all of the investigators came to an agreement on this number.

All the data were normally distributed according to the Shapiro–Wilk test, as *p* values were non-significant at values equal to 0.09, 0.19, and 0.50 for IMW, IPW, and AAW, respectively.

### 3.2. Pont’s Index Reliability

The descriptive statistics are given in Table 1. The mean of IMW was 45.42 ± 2.5, while the mean of Pont’s MW was 47.83 ± 2.8. On the other hand, the mean of IPW was 37.8 ± 2.9, whereas the mean of Pont’s IPW was 38.87 ± 2.3. Finally, the mean of the AAW was 33.65 ± 2.47, but the mean of Pont’s AAW was 36.65 ± 2.5. All the arch widths were overestimated when they were predicted by Pont’s index; in addition, the present study showed a significant difference between Pont’s values in the Kurdish sample for all the anterior, premolar, and molar widths (Table 2).

The Pearson correlations between IMW, IPW, AAW, and SUI were found to be equal to 0.33, 0.32, and 0.52, respectively, which reflect weak positive significant correlations (*p* > 0.05) as described in Table 3. Additionally, the ANOVA test showed a significant relationship between the dependent variables (IMW, IPW, and AAW) and the predictor (SUI); again, this confirms that there was a weak positive correlation between the real values and predicted values from Pont’s index (Table 4). Therefore, new formulas to predict upper arch widths can be developed.

## 4. Discussion

The use of digital technology has grown at an exponential rate in recent years. Intraoral scanners, in particular, have gained interest in orthodontics. It could be the ideal alternative to taking impressions and the future of impressions in orthodontics. These devices provide a wide range of applications with exceptional accuracy, facilitating a more relaxing and efficient clinical experience for both patients and dentists. Virtually all orthodontic appliances may soon be built digitally from imprints taken with intraoral scanners. Medit *i* 700 intraoral scans were used to directly collect all of the data for this study. Since there are no tethered cords, scanning can be conducted from any position, with ease and freedom of movement. The advent of 3D technology, especially intraoral scanners, revolutionized orthodontics by redefining the role of the “traditional” orthodontist in diagnosis and treatment [17].

Numerous studies have investigated the applicability and clinical value of Pont’s index using a variety of selection criteria. The morphology of teeth and the shapes of the face are significantly influenced by racial, cultural, and environmental factors [20,21,22]. In order to assess the reliability of Pont’s Index in the dental class I Kurdish community, the current study focused on this demographic.

The goal of the current investigation was to determine whether there was any link between SUI and the various linear arch widths. The results cannot be compared to research conducted on individuals who have already received orthodontic treatment. Additionally, orthodontic specialists’ understanding of the dental arch changes comes from those that untreated people normally experience during their formative years, which is vital for providing baseline information for treatment planning [23].

According to the study by Defraia et al., many ethnic groups and populations have distinct dental arch sizes and traits [24]. Therefore, it is highly recommended for each ethnic group to have a solid method for predicting the arch measurements.

Pont’s index calculations revealed overestimated values for premolar and molar arch widths, as a smaller arch was seen in the Kurdish community when compared to Pont’s anticipated values.

There was a considerable disparity between the anticipated and measured AAW, IPW, and IMW values. These findings revealed that the expected distance between the molars was higher than the real distance between the molars. This explained why the anticipated values of distance between molars represented wider faces in the Kurdish people, which were not observed in reality. Again, the expected and real distances between premolars and anterior arch widths differed significantly. This explained why the anticipated values of distance between premolars and AAW represented wider faces, which were not observed in reality since the measured values were lower than projected values. These findings are in accordance with previously conducted studies [17,21,22,23,24,25].

On the other hand, the outcomes of the current study are different from the outcomes of other studies such as Gupta et al. [17], who declared a substantial and positive association between the combined maxillary incisal width and premolar as well as molar arch widths.

In a separate study, Shahid et al. developed prediction equations for the estimation of maxillary and mandibular canine and premolar widths from lower incisor and lower first molar widths in a Pakistani population. Nonetheless, Dasgupta’s research on an adult Assamese population discovered a correlation between the steepness of the mandibular plane and the width of the dental arch [26]. It is recommended to utilize personalized archwires based on each patient’s preoperative arch form and width due to the correlation between dental arch width and gender, vertical face morphology, and population groups [27].

The accuracy of Pont’s index in the diagnosis of maxillary transverse discrepancy was tested by Festila et al. in their study. When they compared the Pont’s index results to the CBCT analysis performed at the University of Pennsylvania, they discovered that Pont’s index was suitable for detecting maxillary transverse discrepancy as it had an accuracy rating of 53.28 percent, according to its indicators. On the other hand, evaluation of the maxillary transverse discrepancy on CBCT can be indicated for situations in which the maturation of the midpalatal suture should also be assessed [28]. Hidayati et al. discovered a new equation arch form for the Deutro Malay sub race and recommended a new guidance for the anterior dental arch dimension. The study also found a new arch form of the Deutro Malay sub race. Therefore, the study also underlined that a correct measurement of the dental arch ratio among particular races can be a precondition for effective case planning and treatment in orthodontics and prosthodontics. This is because the ratio of dental arches can vary significantly from race to race [29].

Besides the racial and ethnic differences, this discrepancy between the research studies could be because of the differences in the selection criteria. For instance, one type of occlusion could be included in the inclusion criteria of one study but be excluded in another study. Therefore, according to the results of this study, Pont’s index was proven to be an unreliable tool to predict values for orthodontic treatment in the Kurdish population from a clinical point of view.

Thus, new formulae were established in which coefficients of regression (r) were used to predict anterior, premolar, and molar arch widths by knowing the sum of the maxillary incisor widths. The stepwise regression analysis of the SUI (the dependent variable) that was used to predict the dental arch widths is represented by the following equations:

NEW Formulas:

1. New IMW

Y = 29.73 + 0.5 × X, where Y is the predicted IMW and X is SUI.

2. New IPW

Y = 22.53 + 0.43 × X, where Y is the predicted IPW and X is SUI.

3. New AAW

Y = 12.12 + 0.69 × X, where Y is the predicted AAW and X is SUI.

The coefficients of regression (r) were found to be equal to 0.11, 0.1, and 0.27, respectively. Subsequently, the mean of the real IMW was 45.42 ± 2.5, while the mean of the new IMW (based on the new equation) was 45.28 ± 0.92. On the other hand, the mean of the IPW was 37.8 ± 2.9, whereas the mean of the new IPW was 35.89 ± 0.8. Finally, the mean of the AAW was 33.65 ± 2.47, and the mean of the new AAW was 33.57 ± 1.28. All the arch widths were very close to the real values when they were predicted by the new formulas. Afterwards, a paired *t*-test was used to compare the real and predicted widths (Table 5), which indicated no significant difference between the two values at the *p* > 0.05 level.

Real measurements are the values that were obtained directly from the scanned intraoral image, while expected measurements are the values that were obtained from the predicted equations.

It is clear from the results of the study that the predicted values from the new formulas are close to the real measurements. It is time to build a set of specific equations for every patient as there is no universal or generalized tools or devices that could be used for everyone. As it is clear, with the use of customized appliances, aligners, and digitally guided tools, etc., the revolution in the orthodontic field reflects individualization in each step, starting with the first step of treatment, which is the analysis and interpretation of the arch parameter, arch width, and space analysis.

The equations of the present study differ from previously reported ones because of the differences in the method of measurement, as digitally scanned images and Medit Design software were used instead of calipers. Predicting the dental arch widths in cases of crowded, spaced dentition and class II and III malocclusions is an area that requires more research.

## 5. Conclusions

Pont’s index is not reliable in predicting the arch widths for the Kurdish population; therefore, new formulas were advocated to estimate the inter-molar, inter-premolar, and anterior arch widths. Space analysis, malocclusion treatment, and arch expansion therapy should all take these results into account. Therefore, the derived equations may have further positive effects on diagnostics and treatment preparation.

## Figures and Tables

**Table 1:** Descriptive Statistics.

		*N*	Min	Max	Mean	Std. Dev
Real	IMW	100	40.12	53.53	45.42	2.87
	IPW	100	29.20	41.70	35.78	2.50
	AAW	100	27.45	40.00	33.65	2.48
Pont’s	PIM	100	41.05	52.48	47.83	2.85
	PIP	100	33.35	42.64	38.87	2.32
	PAW	100	31.39	40.13	36.58	2.18

**Table 2:** Paired sample correlations between the real widths and the Pont’s index.

		*N*	Correlation	Sig.
Pair 1	IMW and PIM	100	0.326	0.001 *
Pair 2	IPW and PIP	100	0.315	0.001 *
Pair 3	AAW and PAW	100	0.518	0.000 *

* *p* < 0.05 = Significant.

**Table 3:** Pearson correlation between real widths and SUI.

		Real IMW	SUI	Real IPW	SUI	Real AAW	SUI
Pearson Correlation	IMW	1.000	0.326	1.000	0.315	1.000	0.518
	SUI	0.326	1.000	0.315	1.000	0.518	1.000
Sig. (1-tailed)	IMW, IPW, AAW		0.000		0.001		0.000
	SUI	0.000		0.001		0.000	
N	IMW	100	100	100	100	100	100
	SUI	100	100	100	100	100	100

**Table 4:** ANOVA test shows the relationship between the dependent variables (IMW, IPW, and AAW) and the predictor (SUI).

Model		Sum of Squares	df	Mean Square	F	Sig.
IMW (Dependent variable)	Regression	86.575	1	86.575	11.661	0.001 ^b^
	Residual	727.599	98	7.424		
	Total	814.174	99			
IPW (Dependent variable)	Regression	61.696	1	61.696	10.821	0.001 ^b^
	Residual	558.738	98	5.701		
	Total	620.434	99			
AAW (Dependent variable)	Regression	162.975	1	162.975	35.939	0.000 ^b^
	Residual	444.411	98	4.535		
	Total	607.386	99			

^b^ Predictor: SUI (constant).

**Table 5:** Paired sample tests between real widths and the new equations.

		Mean	Std. Deviation	Std. Error Mean	95% Confidence Interval of the Difference				
					Lower	Upper			
Pair1	IMW-PMW	−2.41	3.32	0.33	−3.07	−1.75	−7.26	99	0.000
Pair2	IMW-New IMW	0.15	2.71	0.27	−0.39	0.68	0.54	99	0.589 *
Pair1	IPW-PIP	−3.08	2.82	0.28	−3.64	−2.52	−10.92	99	0.000
Pair2	IPW-New PIP	−0.12	2.38	0.24	−0.59	0.36	−0.49	99	0.625 *
Pair1	AAW-PAW	−2.92	2.30	0.23	−3.38	−2.47	−12.71	99	0.000
Pair2	AAW-New AAW	0.081	2.12	0.21	−0.339	0.50	0.38	99	0.704 *

* *p* > 0.05 = Significant.

## Data Availability

Data are available from the corresponding author upon request.
